# Green hydrogen generation in alkaline solution using electrodeposited Ni-Co-nano-graphene thin film cathode

**DOI:** 10.1007/s11356-024-32948-0

**Published:** 2024-04-01

**Authors:** Hassan H. Shaarawy, Hala S. Hussein, Adel Attia, Salwa I. Hawash

**Affiliations:** 1 Chemical Engineering & Pilot Plant Department, Engineering Research and Renewable Energy Institute, National Research Centre (NRC), Cairo, Egypt; 2 Physical Chemistry Department, Research Institute of Advanced Materials Technology and Mineral Resources, National Research Centre (NRC), Cairo, Egypt

**Keywords:** Water electrolysis, Electrodeposition, Ni-cobalt electrode, Corrosion, Transmission electron microscopy, Low power consumption

## Abstract

Green hydrogen generation technologies are currently the most pressing worldwide issues, offering promising alternatives to existing fossil fuels that endanger the globe with growing global warming. The current research focuses on the creation of green hydrogen in alkaline electrolytes utilizing a Ni-Co-nano-graphene thin film cathode with a low overvoltage. The recommended conditions for creating the target cathode were studied by electrodepositing a thin Ni-Co-nano-graphene film in a glycinate bath over an iron surface coated with a thin copper interlayer. Using a scanning electron microscope (SEM) and energy-dispersive X-ray (EDX) mapping analysis, the obtained electrode is physically and chemically characterized. These tests confirm that Ni, Co, and nano-graphene are homogeneously dispersed, resulting in a lower electrolysis voltage in green hydrogen generation. Tafel plots obtained to analyze electrode stability revealed that the Ni-Co-nano-graphene cathode was directed to the noble direction, with the lowest corrosion rate. The Ni-Co-nano-graphene generated was used to generate green hydrogen in a 25% KOH solution. For the production of 1 kg of green hydrogen utilizing Ni-Co-nano-graphene electrode, the electrolysis efficiency was 95.6% with a power consumption of 52 kwt h^−1^, whereas it was 56.212. kwt h^−1^ for pure nickel thin film cathode and 54. kwt h^−1^ for nickel cobalt thin film cathode, respectively.

## Introduction

Global energy consumption has recently been continuously increasing as a result of increased living standards and population growth. As a result, the development of renewable energy sources became increasingly vital as environmental pollution and global warming rose. Hydrogen production has opened up significant opportunities for environmental improvement in the transportation industry, which emits no carbon emissions other than water as a byproduct (Dunn 2002).

The obstacles that these technologies face must be solved in order to increase the efficiency of the manufacturing process (Edwards et al. 2008). When compared to conventional solid fuels, which have an energy density of 50 MJ/kg, hydrogen has a high energy density of 140 MJ/kg, making it an appealing energy carrier (Chi and Yu 2018; Dincer 2012). Then, hydrogen gas was developed as an important energy transporter (Zeng and Zhang 2010) and a substantial substitute for conventional crude oil derivative fuels, which directly contribute to environmental pollution.

Going ahead, the expansion of the hydrogen economy is essential (Momirlan and Veziroglu 2005). Hydrogen fuel has various industrial applications, including petrochemical, fuel cell, fertilizer, and petroleum refining processes (Kumar and Himabindu 2019). Hydrogen has been produced using both non-renewable and renewable energy sources, such as fossil fuels. These include the following processes: coal gasification (Burmistrz et al. 2016), biomass (Mujeebu 2016), biological sources (Elsharnouby et al. 2013), water electrolysis (Siracusano et al. 2012), oil/naphtha reforming (Rahimpour et al. 2013), and steam reforming of methane (Xu et al. 2009).

Heat, on the other hand, is employed in biological activities involving bacteria or algae, chemical reactions to liberate hydrogen from organic molecules, and electrolysis to separate water into its component elements, hydrogen, and oxygen. According to (Lee et al. 2018), nonrenewable fossil fuels, particularly methane steam reforming, now produce around 96% of the hydrogen produced worldwide. Fossil fuel-derived hydrogen, however, is less pure and contains more dangerous greenhouse gasses. In order to investigate a low hydrogen overvoltage cathode, an electroconductive base material covered in an alloy layer had to be compromised.

Several elements and a complexing agent from a plating bath were electrodeposited on an electroconductive base material in order to create a low hydrogen voltage cathode (Yamaguchi et al. 1998). It was possible to create a low voltage electrochemical hydrogen producing method by scavenging the anode with sulfur dioxide. The equilibrium voltage required to directly split water into hydrogen and oxygen is lowered when sulfur dioxide is present as a scavenger. This suggests that the free energy has significantly decreased (Mbah et al. 2010). An electroconductive material covered with a layer of cobalt and tin, with the percentage of tin varying from 0.01 to 95% by weight, is used to create a low hydrogen overvoltage cathode (Yamaguchi et al. 1998).

Some researchers deposit nickel alloy or nickel on the exterior of platinum group metal from a bath that contains additives that do not react with the platinum group metal in the plating bath in order to generate a low overvoltage cathode for electrolysis of water or aqueous solutions (Wood 1986). Most research is concentrated on creating cathode materials that are effective in order to reduce the amount of energy used in the process of electrolyzing water to produce hydrogen. While platinum has so far provided nearly wonderful service for the hydrogen evolution process (HER), its scarcity and high cost are drawbacks (Ledendecker et al. 2015). Of all the Ni-based catalysts, Ni-based alloys and hetero-structures with a synergistic neighboring element or domain optimize their surface adsorption capacity, resulting in the most promising electrocatalytic activity and stability.

Finally, some applications for the developed Ni-based HER catalysts are explored, including the chlor-alkali process and microbial electrolysis cells (Gong et al. 2016). By adding foreign elements (Mo, Fe, Cu, etc.) or compounds (rare earths, hydroxides, sulfides, nitrides, etc.) to the earlier systems that show larger surface areas or higher intrinsic activity, the integrated electro-catalytic performance of these alloys is often improved. Even if the fundamental cause of the improvement in electro-catalytic characteristics is not always evident, Mo is frequently added as a supplement to support the primary source of catalytic activity cathode (Yu et al. 2015). Ni doped with transition metals (TM) like iron and cobalt makes for the ideal electrodes for HER in alkaline conditions.

Ni doped with transition metals (TM) like iron and cobalt makes for the ideal electrodes for HER in alkaline conditions. Ni_1+x_Fe_2-x_O_4_ and Ni_1+y_Co_2-y_O nanoparticles in various compositions were investigated. We attribute the similar iso-electric point values (IEP) of all the catalysts to their intrinsic HER catalytic activity. Nevertheless, changing the catalysts’ electrochemical surface area modifies their total catalytic activity. Fractional reaction orders for hydrogen evolution are present in all catalyst compositions and are due to surface acid–base equilibria and double layer effects. Water molecules are known to be adsorbed on the catalyst surface during the first stage of electrolysis and to go through the following reactions in an alkaline solution for the HER process:

1 $$Anode: 2OH^-\to \frac{1}{2}{{\text{O}}}_{2}+2{{\text{e}}}^{-}+ H_2 O$$

2 $$Cathode:2H_2 O+{2{\text{e}}}^{-}\to {{\text{H}}}_{2}+2OH^-$$

The Tafel slope and reaction sequence of the catalysts are congruent with electrochemical adsorption functioning as the rate-determining step in the HER. Electrodes manufactured from catalyst inks containing an anion-exchange ionomer shown lower catalytic activity for the HER than electrodes made from ink containing Nafion. Chronoamperometry revealed that the hydrogen kinetics of NiFe_2_O_4_ and NiCo_2_O_4_ were superior to NiO (Faid et al. 2019). To avoid the high cost of the electrolysis process, numerous researchers developed a low-cost catalyst for the efficient and cost-effective production of hydrogen. Direct creation of hydrogen from air using hygroscopic electrolysis and in situ atmospheric fresh water release powered by wind or solar energy.

The well-known prototype of the process ran continuously for 12 days at approximately 95% Faradaic efficiency. The direct air electrolysis technology, which solves water supply difficulties while producing green hydrogen sustainably and with no environmental impact, may operate in a dry environment with a relative humidity of 4% (Guo et al. 2022). A unique method for co-producing value-added chemicals and evolving hydrogen while conserving energy. This was accomplished by electrochemically supporting total water splitting and producing a current density of 10 mA/cm^2^ using an electro-catalyst with an extremely high energy conversion efficiency and a low cell voltage of 1.39 V (Xu et al. 2022). Alkaline water electrolysis is one of the simplest and most evident processes for producing hydrogen.

Water electrolysis’ principal goals are to reduce energy consumption, expenses, and maintenance while boosting reliability, strength, and safety. Previous research found that using a commercially available graphite electrode at room temperature is a good choice for generating the most hydrogen when electrolyte concentration, applied voltage, and reaction time are all considered (Yuvaraj and Santhanaraj 2014). Commercial electrodes were discovered to be significantly more cost effective than Pt-containing carbon cloth. Because of their low cost and high performance, these electrodes are promising cathode materials for large-scale use in single-chamber microbial electrolysis cells for hydrogen recovery (Farhangi et al. 2014).

The charge transfer coefficient, which is significantly affected by temperature fluctuation, is a major motivator of electrolyzer overvoltage (Tijani et al. 2018). A comprehensive renewable electrolysis system must contain a renewable power source integrated with the electrolysis cell and optimized utilizing interface power electronics to improve performance and reduce costs (Harrison et al. 2010).

When graphene is introduced to pure nickel thin film, the electrical conductivity of nickel and its compounds increases by 15%. (KardanMoghaddam et al. 2020); on the other hand, adding graphene oxide (GO) to the electrodeposition bath, it was possible to significantly increase the electrical conductivity of the nickel thin film produced by jet electrodeposition. Furthermore, it was discovered that the electrical conductivity of the resulting thin film increased as the concentration of GO increased in the electrolysis bath (Ji et al. 2018).

The aim of this work was to create a low overvoltage Ni-Co-nano-graphene thin film cathode for the production of green hydrogen in alkaline electrolytes. The process of electrodeposition involved the incorporation of nanographene as an additive. Nanographene has been injected into the grain boundaries of the nickel–cobalt thin film to enhance grain contact and lower the electrolysis voltage. As a result, less manufacturing power will be needed for the generation of green hydrogen.

## Experimental

### Apparatus

**Fig. 1:**
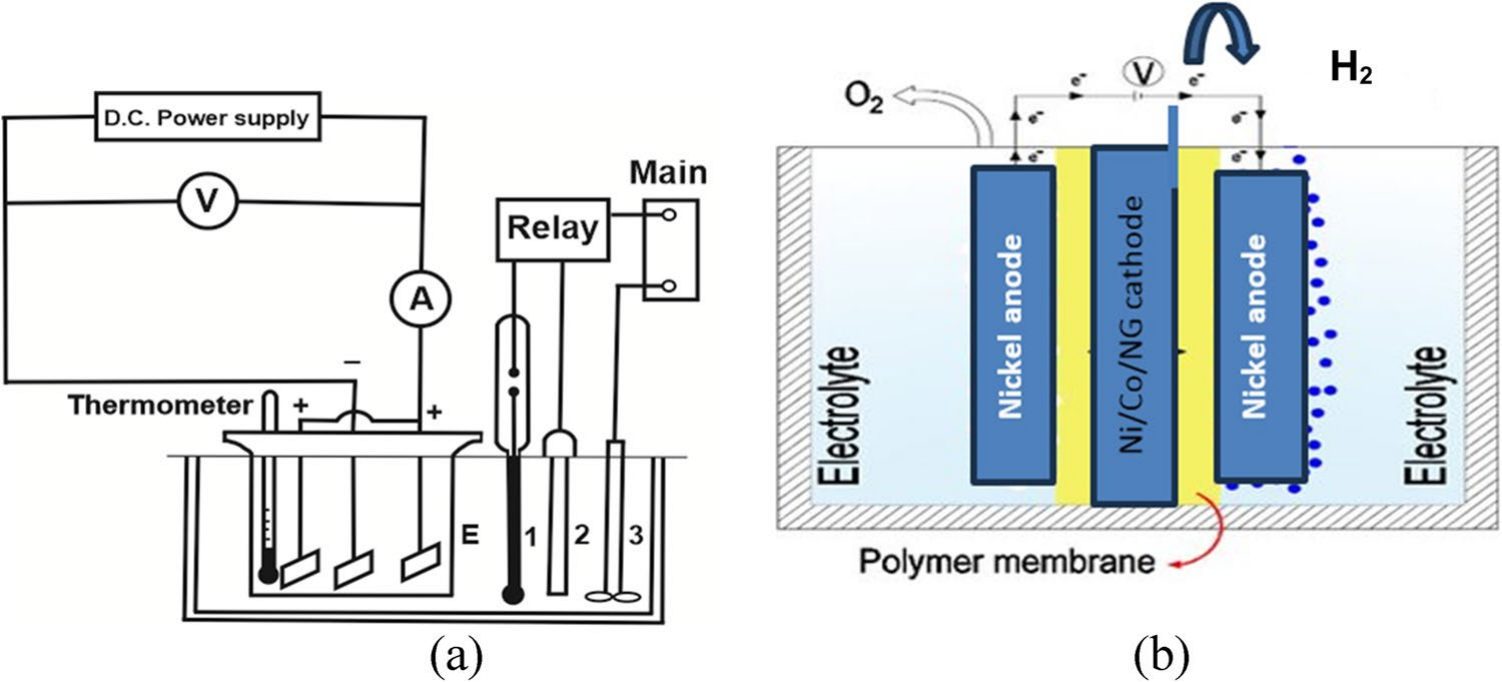
(**a**) Electrodeposition cell used in deposition of Ni-Co-nano-graphene thin film on copper pre-coated steel plate. (**b**) Green hydrogen electrochemical generation and collection cell

In the electrochemical cell represented in Fig. 1(a), Ni-Co-Nano-graphene thin film electrodeposition is performed to prepare the hydrogen generating cathode. It has a capacity of 200 ml and is made of Plexiglas. Two nickel anodes of the highest analytical grade were suspended in the middle of the pathway, and the cathode was a steel plate with an outer area of 10 cm^2^. For supplying direct electric current, a DC power supply of 30 V and 6 A with digital display, voltage, and current control with sensitivity of 0.1 V and 0.1 A was employed. Temperature controller used to modify the electrolysis process between 20 and 100 °C.

### Materials

Carbon steel 4130 (CS) sheets 0.5 mm thick are utilized as the substrate in the electro-deposition process. The specimens were machined to a length of 5 cm and a width of 2 cm. The anode was built of nickel plate (99.5% Ni) with dimensions of 5 cm^2^ cm 0.635 cm supplied by Alfa Aesar, Germany. In the process, a power supply of type GW Instek GPC-3030DQ—Taiwan was utilized, which has a maximum voltage of 30 V and a current of 6 A. The following substances were utilized to make the thin layer Ni-Co-nano-graphene over iron substrate low overvoltage cathode for the electrochemical generation of green hydrogen via alkaline water splitting: nickel chloride, nickel sulfate, boric acid, urea, and cobalt.

Previous work (Hussein et al. 2021) was used to manufacture the nano-metric graphene particles employed in this study. Sigma Aldrich Company provided additional compounds such as EDTA disodium salts and sodium lauryl sulfate (SLS). Before electrochemical deposition of nickel and Ni-Co-nano-graphene thin films over the steel substrate, an interlayer of pure copper thin film was electrodeposited on the steel surface to ensure good adhesion and stability of the nickel cobalt graphene nanoparticle thin film.

The chemical composition of the interlayer copper thin film electro deposition bath was 0.8 M CuSO_4_, 1.5 M H_2_SO_4_, and 1500 Mwt PEG (300 ppm), at 50 mA/cm^2^, room temperature, for 10 min (Vicenzo and Cavallotti 2002), while Table 1 shows the chemical composition of the bath used in the electrochemical deposition of the Ni-Co-nano-graphene thin film over the electrodeposited copper thin film and its working conditions. Boric acid is added to the electrodeposition bath to prevent hydrogen evolution on the nickel and nickel derivatives electroplated film and the formation of extremely high internal stress in the deposited film (Tsuru et al. 2002).

**Table 1:** Bath composition for the electrodeposition of Ni-Co-nanographene thin film and its operating conditions

Code	Component	Concentration	Operating conditions
1	Nickel sulfate	20 g/l	pH 3–9, C.D. 5 mA cm^−2^ − 1300 mA cm^−2^, electrode gap distance 0.5–5 cm, 25 °C, and electrolysis time of 30 to 50 min
2	Nickel chloride	10 g/l	
3	Cobalt chloride	2 g/l	
4	Boric acid	40 g/l	
5	Urea	20 g/l	
6	Glycine	20 g/l	
7	SLS	2 g/l	
8	Nanographene	100–2000 ppm	

### Experimental procedure

To ensure a successful electrodeposition process, the surface of the steel substrate utilized in the fabrication of the low-voltage cathode for hydrogen electrolytic generation must be cleaned and prepared prior to the electrodeposition of copper and Ni-Co-nano-graphene film. To prepare the surface, dip the steel substrate in a concentrated solution of 15% degreaser to remove the layers of fat and oils on the steel’s surface. Table 1 shows the chemical composition and conditions utilized in the process of eliminating oils from the surface of the steel using basic solutions. The steel is then rinsed in running water, and the steel surface is neutralized for 10 s with a dilute 5% solution of nitric acid.

Subsequently, flowing water and distilled water are used to rinse the steel surface. Subsequently, an internal layer of copper was applied by turning on the DC for the desired amount of time on the steel cathode in the copper sulfate deposition bath. After that, the current was turned off; the surface was thoroughly cleaned with running water, distilled water; and copper-coated steel was placed in the Ni-Co-nano-graphene thin film electrodeposition bath. The anode, which was constructed of thin-film steel coated with nickel and copper, was connected to a DC power supply that produced 30 V and 6 A. It also had voltage and current control with sensitivity of 0.1 V and 0.1 A. This allowed for the supply of direct electric current, which was then used to modify the conditions of electrodeposition to the desired level and conduct continuous electric current for the necessary amount of time and intensity.

The finished electrode is then dried using a warm air stream, rinsed with running water, distilled water, and a 50% ethyl alcohol solution before being weighed to find the weight of the layers of copper and nickel alloy that have been deposited. Using a hot, concentrated hydrochloric acid solution, the electrodeposited layer was dissolved. The atomic absorption spectrometer was then used to measure the concentrations of copper, nickel, and cobalt, as well as the nanometric graphene, which allowed Faraday’s law to be applied to determine the electrodeposition efficiency.

To create a Ni-Co-nano-graphene thin film over copper-coated iron substrate with the best possible surface structure and maximum efficiency, the following control operating settings for electrochemical deposition were investigated. The anode–cathode area ratio, electrode gap distance, stirring speed (RPM), electrolysis time, operating pH, electrolysis bath temperature, current density, and, lastly, the impact of the chemical composition of the electro deposition baths were among these operating conditions.

After conducting additional analysis on the obtained electrodeposited film to determine its surface morphology, the thin film was subjected to electrochemical green hydrogen generation from potassium hydroxide solutions. A number of operating conditions were tested to determine the best ones for producing green hydrogen using the suggested cathode, which is appropriate for the alkaline salt used, as well as its concentration, applied current density, electrolysis time, electrolysis temperature, and gap distance between electrodes. It was possible to attain the efficiency of both hydrogen and oxygen generation. Lastly, experimental measurements were made of the suggested electrode's electrolysis potential, corrosion potential, and corrosion current.

### Characterization techniques

The electrodeposited layers’ surface shape and microstructure were shown using JEOL JXA-840A scanning electron microscopy. Transmission electron microscope (TEM) of deposited layer of Ni-Co-nano-graphene was performed using the JEOL-JEM1200 transmission electron microscope. The scratched parts of the Ni-Co-nano-graphene was prepared by adding a distilled water and by the help of ultrasonic a suspension was obtained. Part of this suspension was loaded on a 400-mesh copper grid coated by an amorphous carbon film and let the sample to dry in open air at room temperature. The average diameter of particles was determined by imageJ® (Schneider et al. 2012). Vickers micro-hardness values were measured using the Shimadzu Hardness tester HMV-2t micro-hardness machine under a 970-mN stress. For every specimen, a straight-line average of five values was calculated. Test for linear polarization: The total geometrical surface area of all the working electrodes utilized in this work is 2 cm^2^. Ag/AgCl (3M) served as the reference electrode, and platinum served as the counter electrode. Throughout the entire experiment, 25% KOH was utilized as the electrolyte.

In order to protect the Ag/AgCl reference electrode from the alkalinity of the KOH, a trap holding 3 M KCl was employed to include it during the measurement. A salt bridge was then filled with the test solution (25% KOH). In a traditional three-electrode cell, the electrochemistry analysis of the electrodeposited film on Cu substrate was linked as working electrodes. From the moment the electrode was submerged in the electrolyte, the open circuit potential (OCP) was measured for at least thirty minutes. The high-performance potentiostat/galvanostat instrument, Workstation Autolab PGSTAT302N, is coupled to the electrochemical cell. To obtain the Tafel lines, linear sweep voltammetry was used at a scan rate of 1 mV. By Nova 1.11, the data analysis was completed.

### Hydrogen evolution in alkaline KOH

The electrochemical cell and gas collection system for hydrogen generation are shown in Fig. 1. The resulting thin sheet of Ni-Co-nano-graphene was employed as the cathode between two nickel anodes, using an alkaline membrane separator. In order to produce green hydrogen through alkaline water electrolysis, a number of operating parameters, including electrode gap distance, applied current density, and potassium hydroxide strength, were investigated.

The following formulas for Faraday’s law could be used to determine the current electrodeposition process efficiency, hydrogen production efficiency, oxygen generation efficiency, and water electrolysis efficiency (Strong 1961):

1 $$Z=\frac{m}{Q}=\frac{1}{F}\left(\frac{M}{\nu }\right)=\frac{E}{F}$$

where *M* is the molar mass of the substance and *ν* is the valence of ions. For Faraday’s first 248 law, *M* and *F* are constants, so that the larger the value of *Q* (quantity of electricity) the larger m value. For Faraday’s second law, *Q* and *F* are constants, so that the larger the value of *M*/*v* (equivalent weight), the larger will be *m*. In the simple case of constant-current electrolysis *Q* = *It*, the equation will be as the following:

2 $$m=\frac{It\; M}{F\nu }$$

3 $$n=\frac{It}{F\nu }$$

where *I* is the current in ampere, *t* is the time of electrolysis in seconds, and for thin films of alloys, the equation will be as follows:

4 $$m=\frac{It}{F \times {\sum }_{i}\frac{{w}_{i}\times {\nu }_{i}}{{M}_{i}}}$$

## Results and discussion

### Effect of pH on nickel–cobalt thin film electrodeposition

**Fig. 2:**
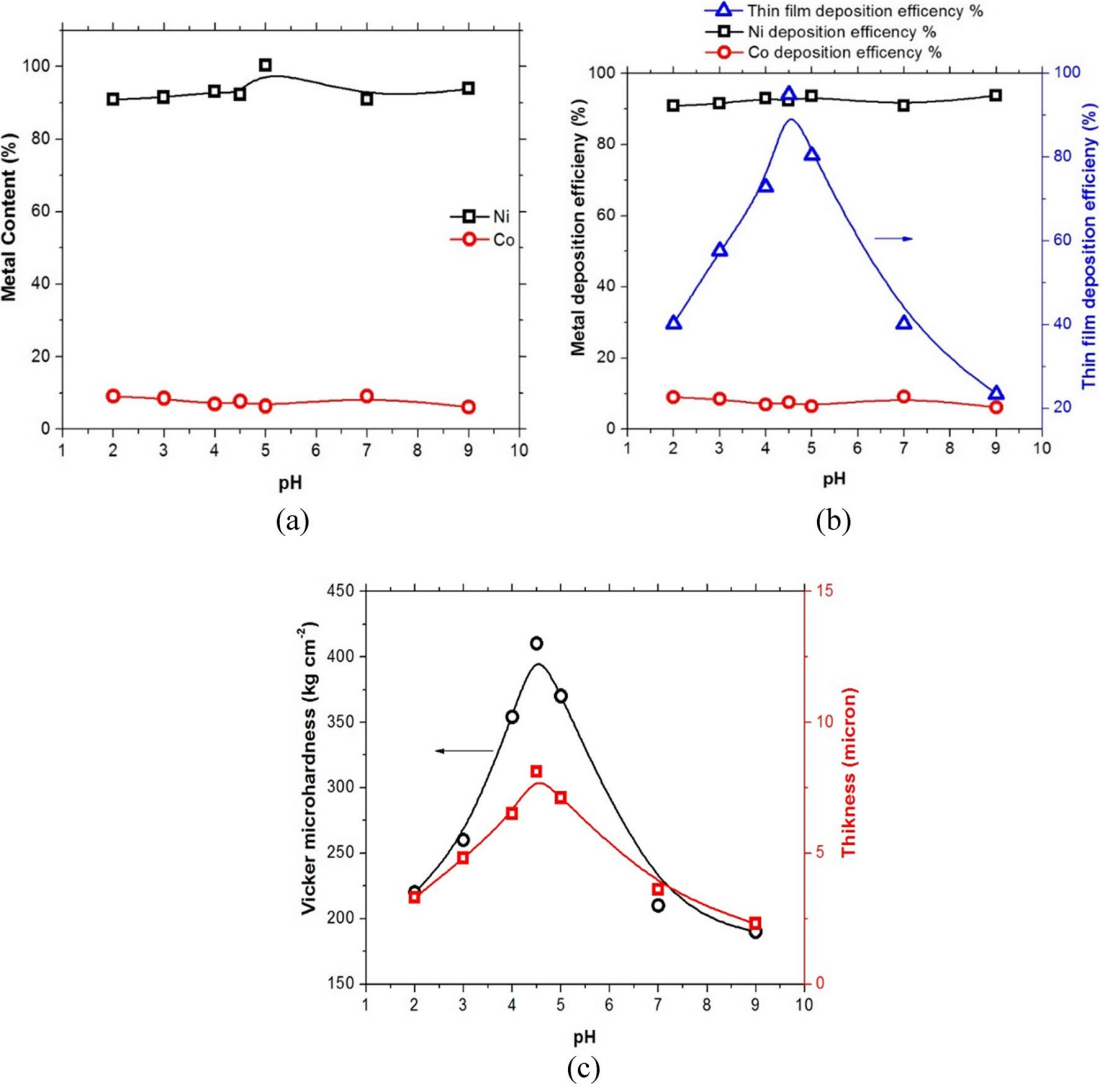
Effect of pH on nickel, cobalt, and nickel–cobalt thin film electrodeposition at operating conditions of C.D. 25 mA cm^−2^, electrolysis time 30 min, 40 °C, 3 cm electrode gap distance, and 2 g/l cobalt chloride, where **a** is metal content in thin film, **b** deposition efficiency, and **c** thickness and Vickers micro-hardness of the obtained thin film

In order to determine the impact of these operating parameters on the metal contents %, deposition efficiency, Vickers micro-hardness, and thin film thickness, the ideal circumstances for the electrodeposition of Ni-Co-nano-graphene were examined. Table 1 listed the bath composition for this electrodeposition method. Figure 2 shows a graphic representation of the bath pH, the most significant operating parameter and how it affects thin film deposition. Anode to cathode area ratio of 2:1, electrode gap distance of 3 cm, electrolysis period of 30 min, and C.D. of 25 mA cm^−2^ were all used throughout the electrodeposition procedure.

The findings indicate that within the used pH range, the nickel content ranged from 90.9 to 93% and the cobalt content from 9 to 6%. Meanwhile, the thin film deposition efficiency for Ni, Co, and Ni-Co was found to be 90–93.8% for Ni, 2–6% for Co, and 40–23% for thin film, respectively. At pH 2, the Vickers micro-hardness was 220 kg/cm^2^. At pH 4.5, it rose to 410 kg/cm^2^, and at pH 9, it began to decline to 190 kg/cm^2^. Along with the pH increase, the thin film thickness likewise declined from pH 2 to pH 4.5, reaching 2.3 μm at pH 9. Values ranged from 3.3 to 8.1 μm.

Low pH levels result in hydrogen evolution due to pH variation, which lowers current efficiency. The agitation of the bath solution caused by the evolution of hydrogen at these low pH levels results in a thin coating that adheres well and has a high micro-hardness rating. The addition of ammonia causes the pH to rise, which in turn causes hydrogen evolution to decrease and current efficiency to increase. This process reaches its peak at pH 4.5. Further pH increases cause the formation of fine particles of nickel hydroxide, which results in an unstable thin film with low micro-hardness values. On the other hand, and due to the development of nickel and cobalt glycinate complexes at low pH values and high hydrogen evolution at the cathode, there was strong polarization of both nickel and cobalt in the deposited thin film. This resulted in the formation of a stable, brilliant, and well adherent nickel–cobalt thin film. High pH values (over 6) cause the solution at the cathode to become alkaline due to a decrease in hydrogen ions, which causes hydroxides and basic salts to be included in the deposits. These high pH levels can also cause oxides to develop and be included in the deposit (Young and Egerman 1937).

### Effect of current density on nickel–cobalt thin film electrodeposition

**Fig. 3:**
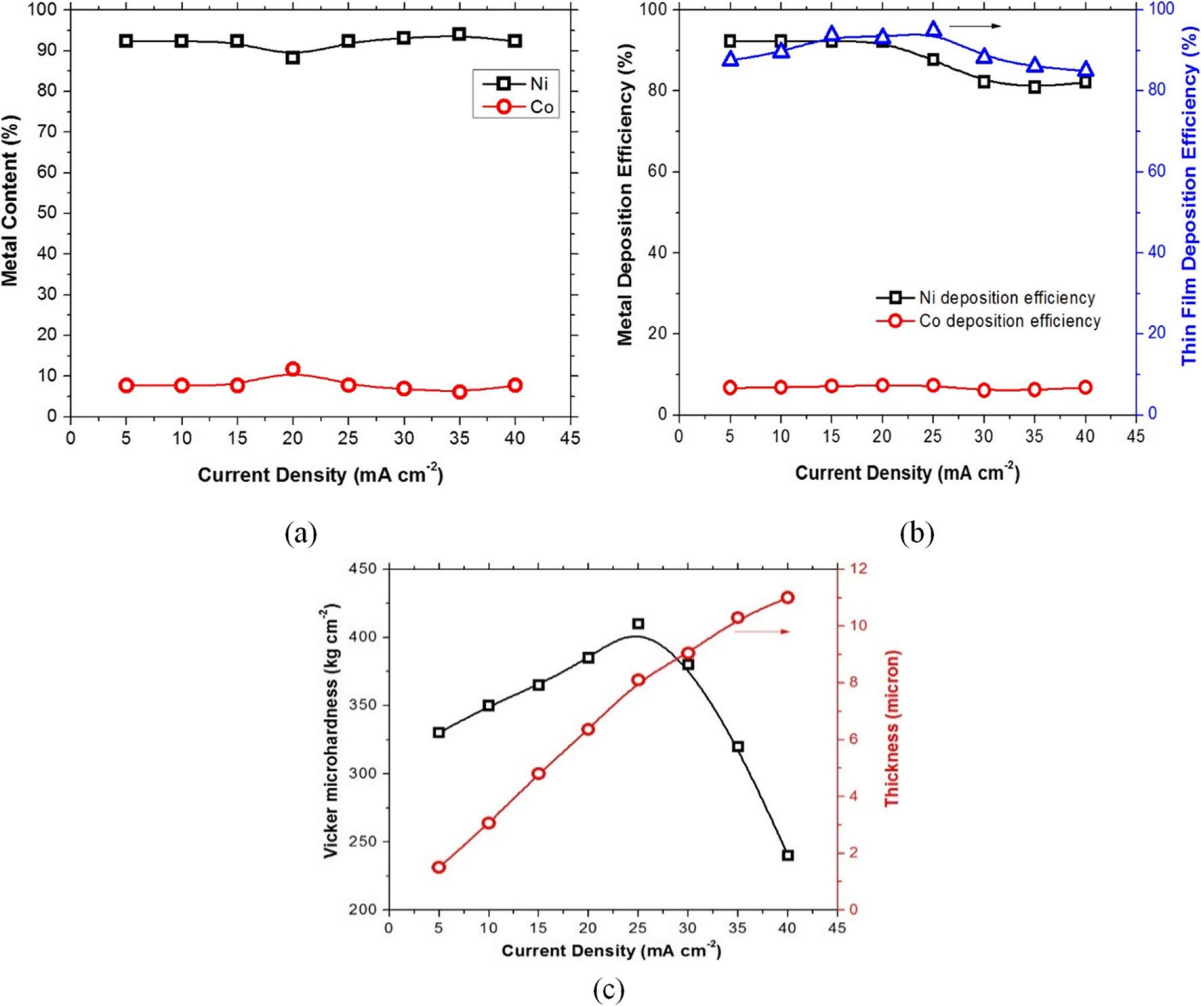
Effect of applied current density on electrodeposition of nickel, cobalt, and nickel–cobalt thin film at operating conditions of pH 4.5, electrolysis time 30 min., 40 °C, 3 cm electrode gap distance, and 2 g/l cobalt chloride, where **a** is metal content in thin film, **b** deposition efficiency, and **c** thickness and Vickers micro-hardness of the obtained thin film

Figure 3 shows a visual representation of the influence of the applied current density on the Ni-Co electrodeposited thin film formed at pH 4.5, electrolysis time 30 min, 40 °C, electrode gap distance 3 cm, and anode to cathode area ratio 2:1. According to the results, the thickness of the obtained film increased as the applied current density increased, but with unstable and non-adhered thin film with black oxide film as the applied current density increased from 30 to 40 mA/cm^2^. As a result, 25 mA/cm^2^ was selected as the recommended deposition current density. The Ni, Co metal content in the thin film composition and the thin film deposition efficiency% have no significant change. The Vickers micro-hardness increased from 330 kg/cm^2^ at 5 mA/cm^2^ to 410 kg/cm^2^ at 25 mA/cm^2^, then decreased to 240 kg/cm^2^ at 40 mA/cm^2^.

The variation in thin film compositions and current efficiencies with changing current density can be attributed to either agitation caused by gas evolution or the rapid deposition of complexed cobalt ions on the cathodic double layer, which becomes difficult under diffusion control at high current densities.

### Effect of electrolysis time on nickel–cobalt thin film electrodeposition

At working conditions of pH 4.5, electrolysis time of 30 min, applied current density of 25 mA/cm^2^, 40 °C, electrode gap distance of 3 cm, and anode to cathode area ratio of 2:1, the impact of electrodeposition time on the characteristics of the Ni-Co produced thin film was investigated. The data obtained indicate that while the Ni% content declined marginally and the Co% content slightly increased with an increase in electrolysis duration, the thin film deposition efficiency dramatically reduced, reaching its lowest value of 87% at 60 min of electrodeposition time. Vickers micro-hardness declined with increasing electrodeposition time because the resulting thin layer is unstable and non-adherent, yet thickness increased and reached 12 microns after 60 min. The most stable and adhered Ni-Co thin film was obtained at 30 min of electrodeposition time which selected as recommended.

Bath temperature has no effect on Ni%, Co%, metal content, or deposition efficiency; however Vickers micro hardness achieves its maximum at 40 °C; hence, this temperature was chosen as the best bath temperature. In addition, the effect of cobalt chloride concentration on the resulting Ni/Co thin film deposited at 25 mA/cm^2^, pH 4.5, 30 min. electrolysis time, 40 °C, 3-cm gap distance, and anode to cathode area ratio of 2:1 was investigated. Indicates that, despite increasing cobalt chloride concentrations, the Ni% content remains the dominant proportion, the cobalt content is minor, and the film deposition efficiency somewhat drops.

The results show that when the cobalt chloride concentration increases, the Vickers micro-hardness declines dramatically, from 450 kg/cm^2^ at zero cobalt chloride concentration to 385 kg/cm^2^ at 10 g cobalt chloride concentration. The most suitable cobalt concentration is 2 g/l, with a deposition efficiency of 95% and a Vickers micro-hardness of 410 kg/cm^2^ with a metallic look, stable, and well adherent thin coating; thus, this concentration was chosen. The effect of nano-graphene additives on the properties of the obtained Ni-Co thin film deposits was investigated at electrodeposition operating conditions of pH 4.5, C.D 25 mA cm^−2^, 30 min. electrolysis time, 40 °C, 3 cm gap distance, anode to cathode area ratio 2:1, and 2 g/l cobalt chloride concentration.

The results reveal that when the nanographene concentration grows, the Ni% and Co% metal content declines and the nanographene% content slightly increases, while the thin film deposition efficiency decreases dramatically, reaching a low of 51% at 2000 mg/m^2^. The Vickers micro-hardness and thin film thickness, on the other hand, drop as the nanographene concentration increases, reaching their lowest values at 2000 mg/l nano-graphene, which are 170 kg/cm^2^ and 4.2 μm, respectively.

The most acceptable deposition efficiency of 88% and Vickers micro-hardness of 395 kg/cm^2^ were obtained at 250 mg/l nanographene with film thickness of 7.47 μm; hence, the concentration of 250 mg/l nanographene was chosen as optimal. Based on the aforementioned results, the optimal electrodeposition parameters for Ni-Co-nanographene thin film electrodeposition were pH 4.5, C.D 25 mA/cm^2^, electrolysis time 30 min, at 40 °C, with 3-cm electrode gap distance and anode to cathode area ratio 2:1 utilizing electrodeposition bath.

It is concluded that glycine, in the presence or absence of ammonium hydroxide (required to adjust the pH value of the bath), acts as a complexing agent, resulting in the formation of corresponding glycinate and ammonia complexes, which facilitate the cathode reaction and allow both Ni^2+^ and Co^2+^ to be reduced simultaneously at the deposition conditions. Urea functions as a stabilizing agent, increasing the bath’s leveling capacity and preventing the turbidity of the used electrolyte. The use of boric acid not only suppresses hydrolysis and prevents pitting formation, but it also widens the current density ranges over which good-quality deposits can be formed by eliminating the burnt and black look at higher current densities (Horkans 1981).

In addition to the presence of formed ammoniacal and nickel glycinate ((Arnold et al. 1985; Oraby et al. 2023)), which stabilize pH values and increase the hardness of the obtained thin film, the bath’s buffering capacity is extended across the entire range of pH values used.

Due to the special properties of graphene and its derivatives, graphene oxide and graphene were combined to create an affordable high electrocatalytic oxygen evolution electrode for hydrogen synthesis via water splitting (Babar et al. 2020).

**Fig. 4:**
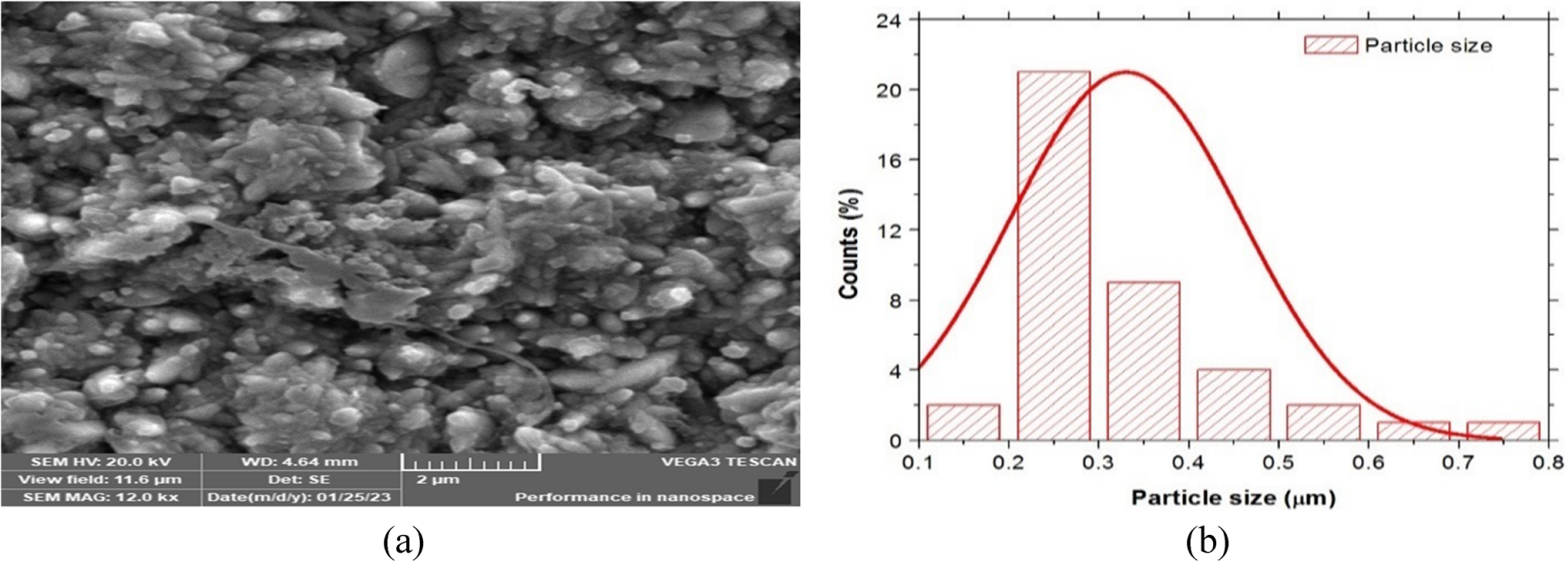
(**a**) SEM analysis nanostructure Ni-Co-nano-graphene thin film electrodes obtained at the recommended conditions for electrodeposition on copper coated steel substrate. (**b**) The histogram of the obtained thin film of Ni-Co-nano-graphene nanostructure substrate

The SEM characterization of the coat produced by electrodepositing a thin layer of Ni-Co-nano-graphene over a steel substrate coated in copper is displayed in Fig. 4a. The resulting thin film has a cauliflower-like particle arrangement, as shown in Fig. 4a, and its grain size ranges from 200 to 800 nm. The histogram of the thin film of Ni-Co-nano-graphene nanostructure electrode that was developed is shown in Fig. 4b. Furthermore, the arrangement of the particles in Fig. 4b shows that there is a high active outer surface area, as evidenced by the 3D particle arrangement. This large area permits the consumption of a high ampere during electrolysis in alkaline water, which results in the generation of a significant amount of created green hydrogen.

**Fig. 5:**
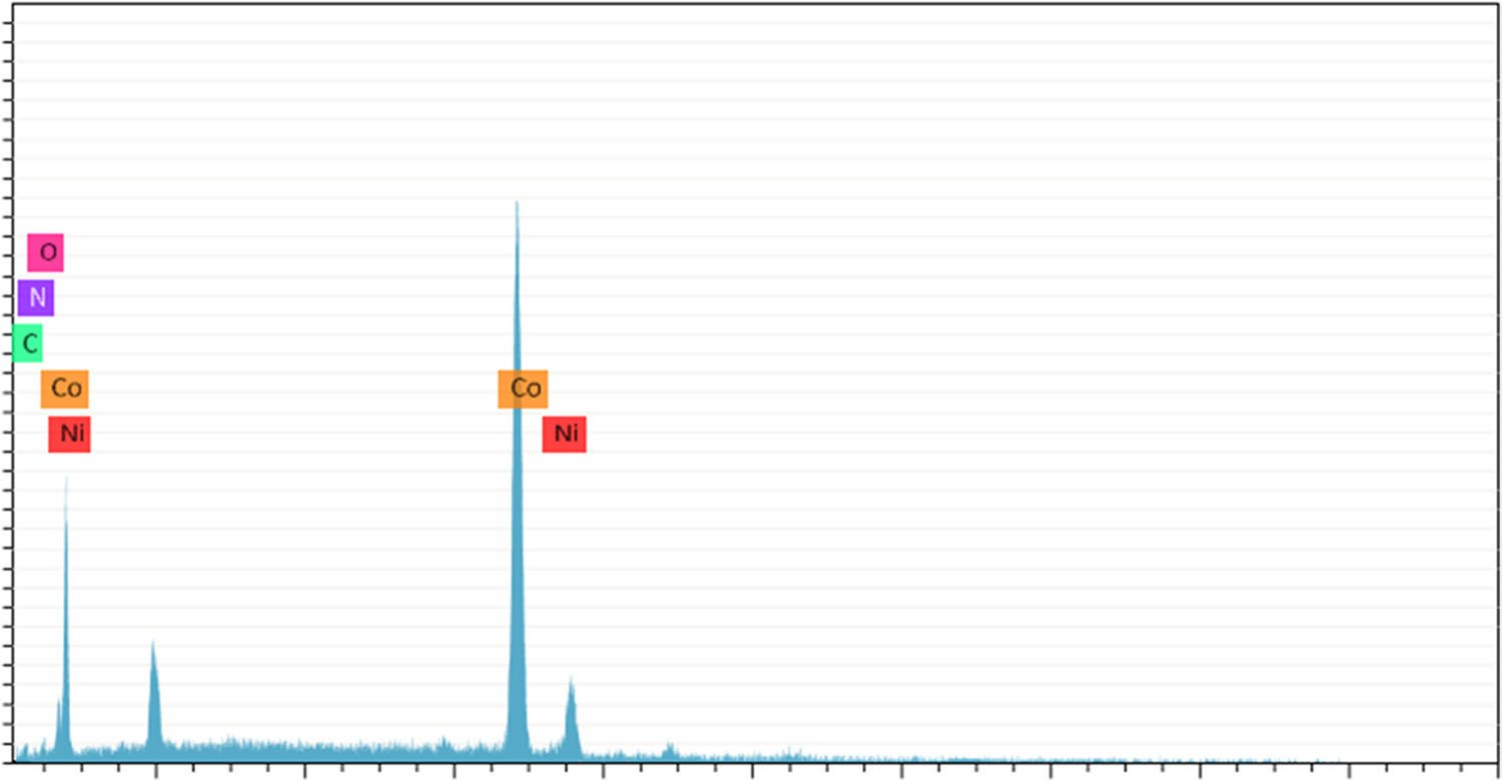
EDX analysis nanostructure Ni-Co-nano-graphene thin film electrodes obtained at the recommended conditions for electrodeposition on copper coated steel substrate

**Fig. 6:**
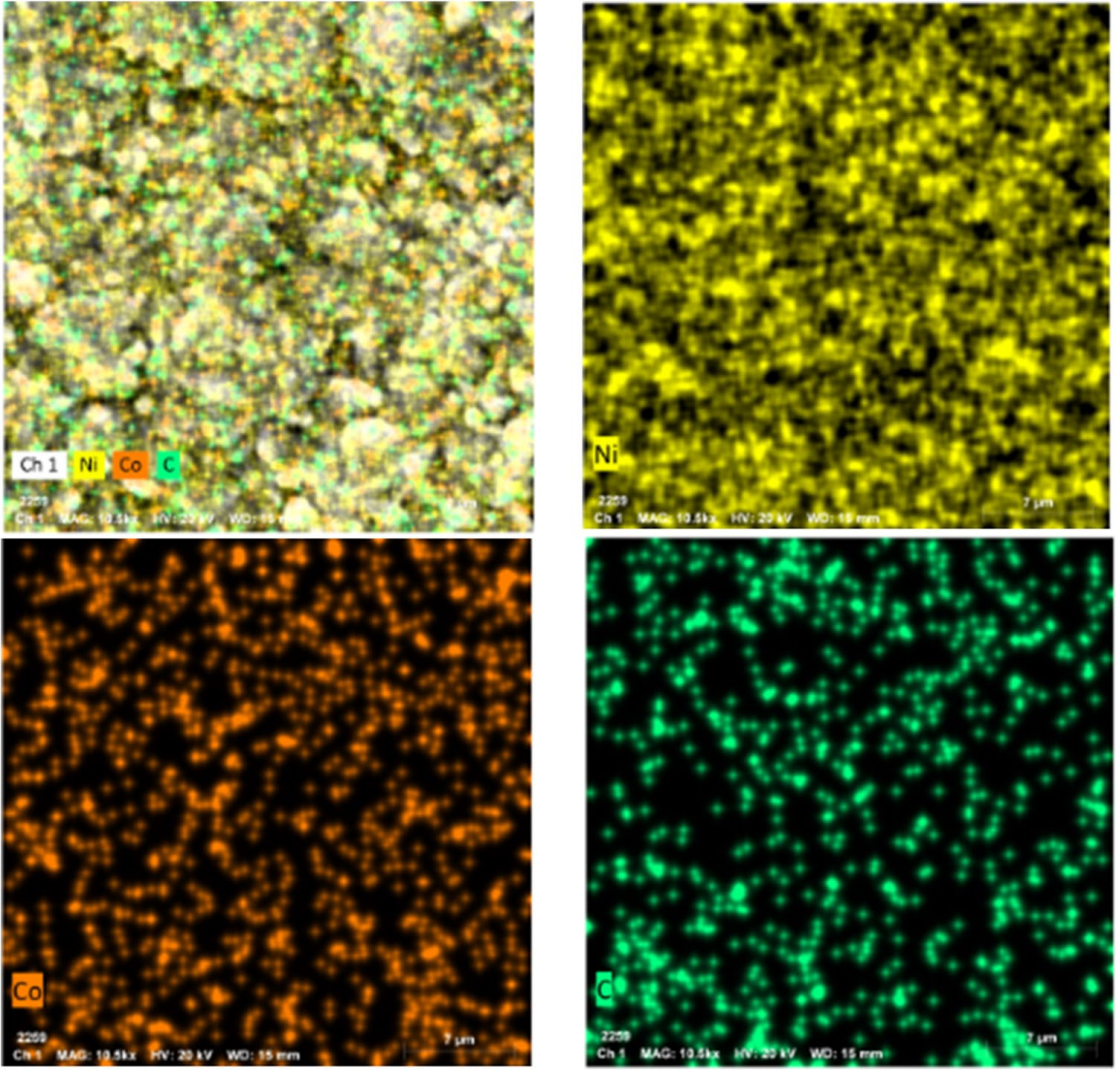
Energy dispersive X-ray (EDX) mapping analysis of composites Ni-Co-nano-graphene

Based on EDX examination, the composition of the Ni-Co-nano-graphene thin film is 94.33%, 4.59%, and 0.42% for nickel, cobalt, and nano-graphene, respectively, as shown in Fig. 5 and Table 2. These outcomes also line up with the SEM–EDX mapping pictures displayed in Fig. 6. Moreover, these pictures verify the uniform dispersion of Ni, Co, and nano-graphene. Figure 6 clearly shows that the cobalt and nano-graphene particles are very well dispersed throughout the nickel thin film. This could result in increased electrical conductivity throughout the thin film, which would decrease the electrolysis voltage and raise the amperage while using the same amount of electric power, increasing the amount of hydrogen that is generated.

**Table 2:** EDX nanostructure analysis of Ni-Co-nano-graphene thin film electrodes developed under ideal conditions

Element	At. no	Mass (%)	Mass norm. (%)	Atom (%)	Abs. error (%)	Rel. error (%)
Nickel	28	32.79	94.33	94.33	0.95	2.88
Cobalt	27	0.11	4.59	4.59	0.04	23.96
Oxygen	8	0.06	0.66	0.66	0.08	133.92
Carbon	6	0.04	0.42	0.42	0.00	10.00

**Fig. 7:**
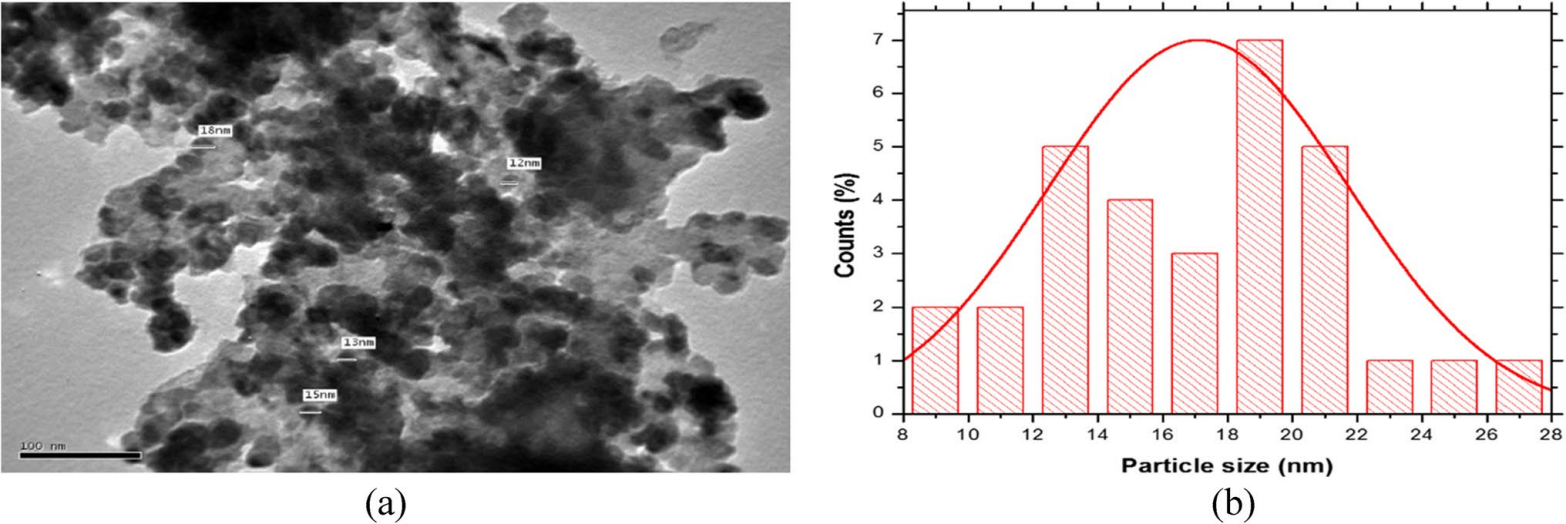
(**a**) TEM analysis of the nanostructure Ni-Co-nano-graphene thin film electrodes obtained at the recommended conditions for electrodeposition on copper coated steel substrate. (**b**) Particle size distribution of the nanostructure Ni-Co-nano-graphene thin film electrodes obtained at the recommended conditions for electrodeposition on copper coated steel

From the EDAX analysis and particle size distribution, it was concluded that the majority of the Ni grains are deposited in microstructures with a grain width of roughly 250 nm and a percentage of 21%. According to the mapping results, the nanoforms of cobalt and nanographene were primarily deposited between the grain boundaries of nickel. TEM on the other hand reveals grain boundaries of the nickel microstructure grains through the electrodeposited Ni-Co-nano-graphene thin film. Figure 7(a) displays the thin film’s TEM photograph, while Fig. 7(b) displays the particle size distribution.

TEM analysis makes it evident that the deposited particles are semicircle nanoform, with a majority of particles ranged diameter of approximately 2 nm and a percentage of 7%. In order to produce green hydrogen, the cathode’s service life, efficiency in producing hydrogen during electrolysis, resistance to high current densities, and power consumption for hydrogen generation are the most crucial factors to consider. The liner polarization test will be performed and examined in the next part in order to determine the corrosion rate, corrosion voltage, corrosion current, and service life duration.

### Linear polarization and electrode service life

5 $${\text{Ni}}{\left({\text{OH}}\right)}_{2}+{{\text{OH}}}^{-}\to {\text{NiOOH}}+{{\text{H}}}_{2}{\text{O}}+{{\text{e}}}^{-}$$

6 $${\text{Ni}}+{{\text{H}}}_{2}{\text{O}}\rightleftharpoons {\text{NiO}}+2{{\text{H}}}^{+}+2{{\text{e}}}^{-}$$

7 $$3{\text{NiO}}+{{\text{H}}}_{2}{\text{O}}\rightleftharpoons {{\text{Ni}}}_{3}{{\text{O}}}_{4}+2{{\text{H}}}^{+}+2{{\text{e}}}^{-}$$

8 $$2{\text{NiO}}+{{\text{H}}}_{2}{\text{O}}\rightleftharpoons {{\text{Ni}}}_{2}{{\text{O}}}_{3}+2{{\text{H}}}^{+}+2{{\text{e}}}^{-}$$

**Fig. 8:**
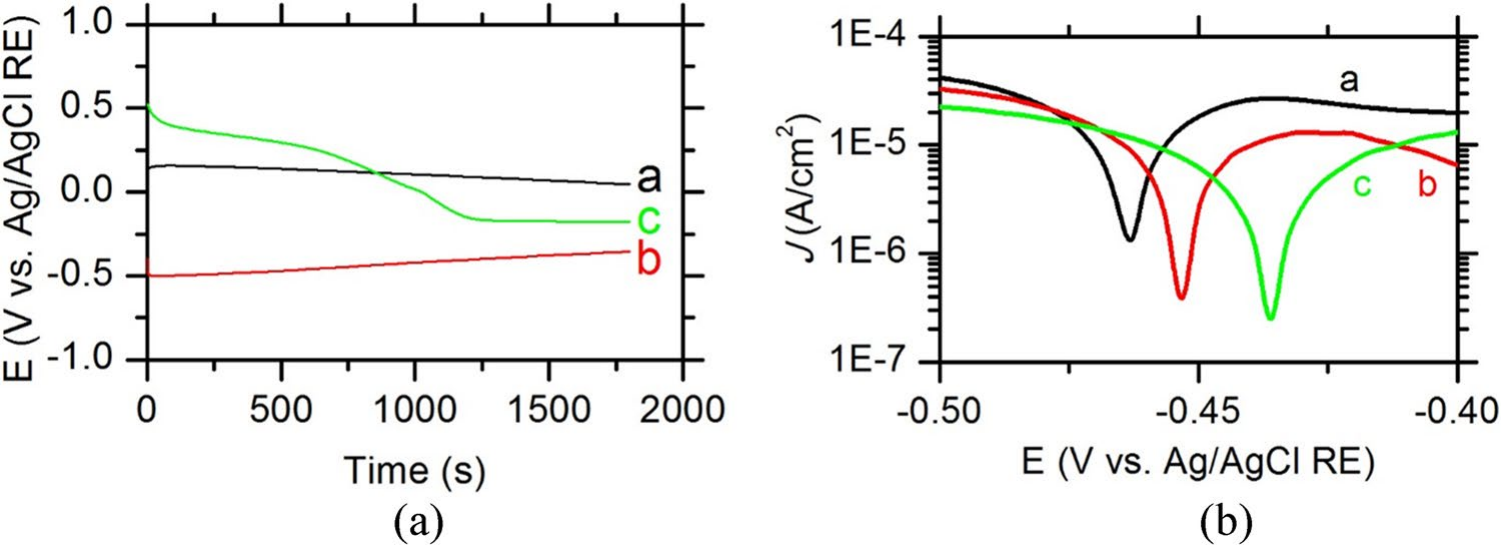
(**a**) Open circuit potential of thick film electrode materials fabricated by electrodeposited thick films on Cu substrate of Ni (a), Ni-Co (b), and Ni-Co-nano-graphene (c). Electrodes were investigated in 25% KOH. (**b**) Tafel plots of thick film electrode materials fabricated by electrodeposition on Cu substrate of (a), Ni-Co (b), and Ni-Co-nano-graphene (c). Electrodes were investigated in 25% KOH

Figure 8(a) depicts the open circuit potential (OCP) showed that Nickel electrodeposited on Cu substrate has a steady state after 30 min of immersion in 25% KOH. This electrode showed a shift in OCP towards the positive direction; the steady state reached (46 mV vs. Ag/AgCl RE, see curve a) indicated that the electrode was going to the noble direction. This was understood as the chance of the formation of nickel hydroxide in alkaline medium to form hydrated nickel oxide which in turn converted to nickel oxides according to the following equations: The NiOOH can be converted to NiO which can undergo further reaction to form hydrogen as a result of water reduction, and the NiO can be converted to higher oxidation state (from + 2 to + 2.67 and + 3) as explained in Eqs. 6 and 7

The exact oxidation of the formed nickel oxide in this work was not determined. However, when the Co was added to the Ni in the electrodeposition (Ni-Co on Cu substrate), the OCP showed a steady state (-355 mV vs. Ag/AgCl RE) with a gradient slope towards the positive direction. However, this was never reaching the same steady state of the Ni electrodeposited on Cu within the time limit of the OCP (see curve b).

The addition of graphene to the electrodeposited film on Cu (Ni-Co-nano-graphene) showed a steady state midst the Ni and Ni-Co on Cu substrate. However, this behavior was showing a two-step pattern started from the far most positive shift until it reached steady state of 175 mV vs. Ag/AgCl RE that lie between the other synthesized electrodeposited electrodes. It is worth to mention that the OCP is showing the electrochemical behavior of the outer layer which need to have long time to reach the actual behavior of the electrode. This disadvantage was overcome by other techniques such as linear sweep voltammetry to get the Tafel lines (Attia and Abdel-Fatah 2020).

Tafel lines of electrodeposited thin film was carried out in 5% KOH was depicted in Fig. 8(b). The *I*_corr_ was decreased in the following order: Ni-Co-nano-graphene < Ni-Co < Ni electrodeposited materials on Cu.

The corrosion rate will be affected in the same order as shown in Table 3. The corrosion rate was the highest for Ni electrodeposited on Cu while it was the lowest for the Ni-Co-nano-graphene electrodeposited on Cu. On the other hand, the polarization resistance increased in the following order: Ni-Co-nano-graphene > Ni-Co > Ni electrodeposited on Cu in 25% KOH (from ~ 476 to ~ 1625 Ω). This meant that the oxidation resistance was higher in that order. In the sense of using these materials in H_2_ production, the addition of the Co and graphene showed higher resistance to corrosion among the other electrodeposited material on Cu substrate which means that there was an enhancement of the electrodeposited material in resisting corrosion during the H_2_ production. With respect to the obtained thickness of the Ni-Co-nanographene film over copper coated iron substrate which is 8 μm, the corrosion rate of the obtained film at recommended electrodeposition process and considering the assumption of replacing the electrode when the film thickness decreased to its 40% value during the green hydrogen production process; it is found that the electrode life time may be 2 years and the electrode life time could be increased to 5 years by increasing the electrodeposition time to 120 min instead of 60 min. which means that the obtained thin film thickness will be 25 μm. Besides the corrosion rate and electrode service life time, the hydrogen production operating conditions and its process efficiency will be investigated in the following section.

**Table 3:** Corrosion parameters for thick film electrode materials by electrodeposition on Cu in 25% KOH

	*ß*_*a*_ V dec^−1^)	*ß*_*c*_ (V dec^−1^)	*E*_corr_ (V)	*j*_corr_ (A cm^−2^)	Corrosion rate (mm year^−1^)	Polarization resistance (ohm)
Ni	0.02875 ± (0.00355)	0.02914 ± (0.01422)	− 0.46354 ± (0)	6.251 × 10^−6^ ± (1.189 × 10^−6^)	0.13498 ± (0.02552)	476.16333 ± (30.69805)
Ni-Co	0.02011 ± (0.01273)	0.02355 ± (0.01725)	− 0.4534 (0)	2.657 × 10^−6^ ± (1.032 × 10^−6^)	0.05731 ± (0.02227)	713.75667 ± (236.46908)
Ni-Co-nano-graphene	0.02379 ± (0.01027)	0.03186 ± (0.00472)	− 0.43632 ± (0)	1.775 × 10^−6^ ± (2.813 × 10^−7^)	0.03834 ± (0.00611)	1625.16667 ± (321.76072)

### Hydrogen electrolytic generation

The target of this study is to prepare highly conductive Ni-Co-nano-graphene thin film deposited on still coated copper substrate with low overvoltage to be used as a cathode for green hydrogen generation via KOH alkaline solution electrolysis. Several operating conditions during water electrolysis are very effective in green hydrogen generation efficiency such as electrode type, potassium hydroxide electrolyte composition, applied current density, electrodes gap distance, and electrolysis time. In the following, the effect of these operating parameters will be explained.

### Effect of electrode type

The production of green hydrogen using nickel thin film, nickel cobalt thin film, and nickel/cobalt/nano-graphene thin film cathodes was compared. The electrolysis efficiency of the Ni-Co-nano-graphene electrode was found to be the greatest, reaching 95.6% under operating conditions of 700 mA/cm^2^, 60 min of electrolysis duration, and a 0.5-cm gap spacing. The nickel thin film cathode and the nickel cobalt thin film cathode have electrolysis efficiencies of 87% and 93%, respectively.

For the Ni, Ni-Co, and Ni-Co-nanographene cathodes, the observed voltage values during electrolysis were 1.9 V, 1.852 V, and 1.75 V, respectively. It is evident that adding traces of nano-graphene to nickel–cobalt thin films reduces the electrolysis voltage needed to produce green hydrogen from 1.9 to 1.75 V. As a result, less power is needed to generate hydrogen for nickel thin-film cathodes that do not contain nano-graphene additives. With a 3% increase in hydrogen generation efficiency, the power consumption decrease with the Ni-Co-nano-graphene cathode is around 8.1% lower than with the nickel case. The above nickel, cobalt, and nano-graphene data will be used to determine which film cathode should be used to produce green hydrogen.

### Effect of potassium hydroxide concentration

**Fig. 9:**
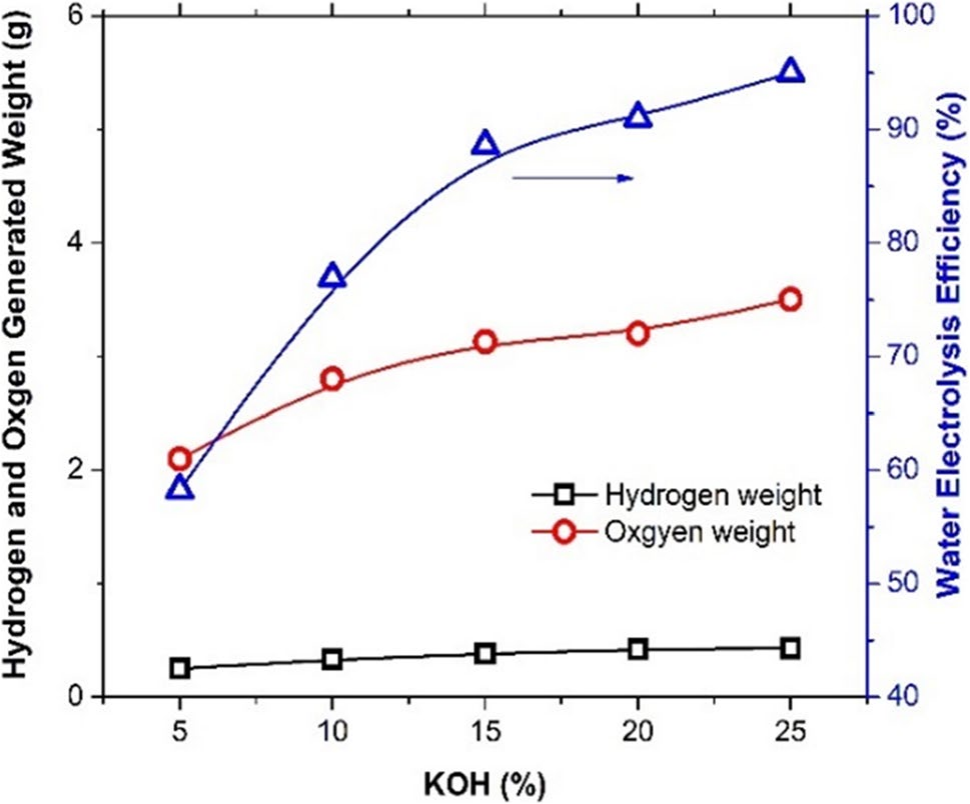
Effect of potassium hydroxide composition on the water electrolysis efficiency, hydrogen generation, and oxygen generation at using Ni-Co-nano-graphene cathode, C.D. 700 mA/cm^2^, 0.5-cm electrode gap distance, and 60-min electrolysis time

Figure 9 shows the graphically displayed electrolysis efficiency, hydrogen and oxygen electrolytic generation at 700 mA/cm^2^, 0.5-cm electrode gap density, and 60-min electrolysis time as a function of the composition of the potassium hydroxide electrolyte. This electrolyte composition was determined to be optimal because the results show that as KOH strength increases, the generated hydrogen and oxygen reach their highest values at 25% KOH concentration. Additionally, the water electrolysis efficiency increases and reaches 95.6% at 25% KOH.

### Effect of applied current density

**Fig. 10:**
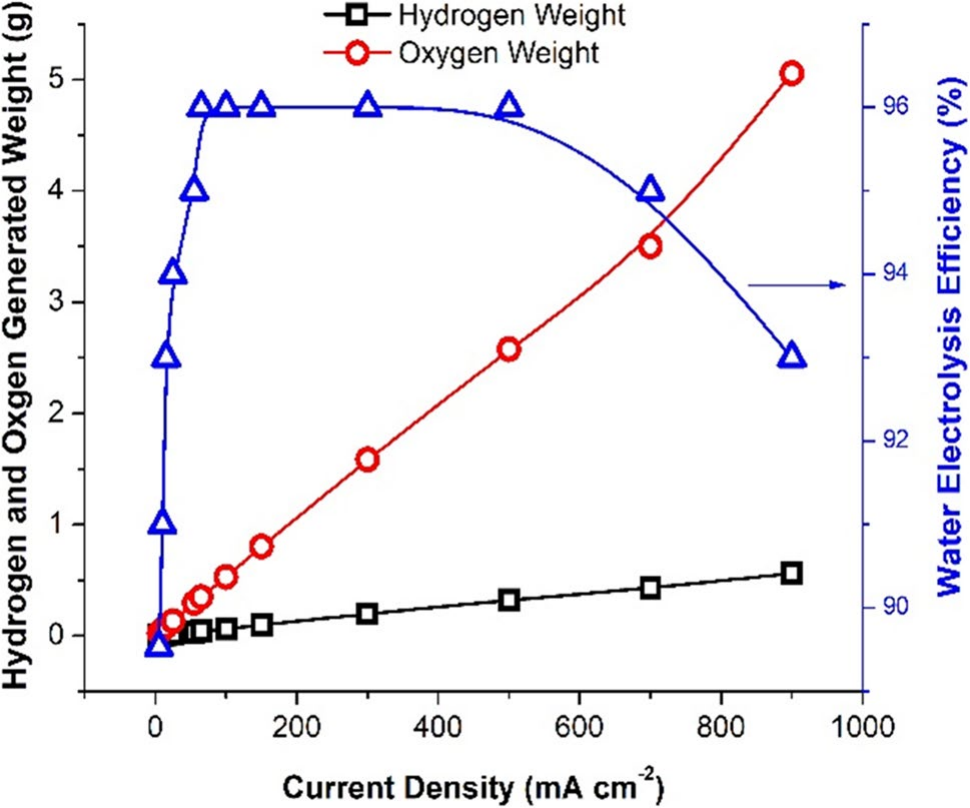
Effect of applied current density on the water electrolysis efficiency, hydrogen generation, and oxygen generation at using Ni-Co-nano-graphene cathode, at 25% KOH, 0.5 cm electrode gap distance, and 60 min. electrolysis time

Figure 10 depicts the influence of applied current density on electrolysis efficiency, hydrogen, and oxygen electrolytic generation while employing a Ni-Co-nano-graphene cathode in 25% KOH with a 0.5-cm electrode gap density for 60 min.

The results show that as the applied current density increases, so does the amount of hydrogen and oxygen produced. While water electrolysis efficiency rises to 95% at 600 mA/cm^2^ and subsequently begins to fall, a current density value of 700 mA/cm^2^ was chosen as optimal in terms of the amount of green hydrogen and oxygen created.

Hydrogen and oxygen electrolytic generation using a Ni-Co-nano-graphene cathode in 25% KOH, 700 mA/cm^2^, for 60 min reveals that as the gap distance between cathode and anode increases, so does the amount of hydrogen and oxygen generated and the electrolysis process efficiency, so a gap distance of 0.5 cm was chosen as optimal.

The influence of electrolysis time on hydrogen, oxygen, water electrolysis efficiency, and the stability of the Ni-Co-nano-graphene cathode was also investigated. With consistent water electrolysis current efficiency and up to 50 h, it was discovered that as the electrolysis time grows, so does the weight of the created green hydrogen and oxygen. During the electrolysis period, there was no change in the weight of the employed cathode, its coated thin film look, or adhesion. Based on the foregoing, the produced electrode performs well at recommended circumstances of 700 mA/cm^2^, 25% KOH, gap distance 0.5 cm, and 95% electrolysis efficiency, producing green hydrogen via the following mechanism: 

In water electrolysis, HER is the primary half-reaction that produces hydrogen at the cathode via a two-electron transfer pathway. The mechanism of this HER is extremely reliant on environmental conditions. In acidic medium, there are three probable reaction steps for the HER reaction.

9 $$\mathrm{H }\;(1{\text{a}}) + {e}^{-} = {{\text{H}}}_{{\text{ad}}}$$

10 $$\mathrm{H }\;(1{\text{b}}) + {e}^{-} + {{\text{H}}}_{{\text{ad}}} = {{\text{H}}}_{2}$$

11 $${2{\text{H}}}_{{\text{ad}}} = {{\text{H}}}_{2}$$

To generate adsorbed hydrogen, the initial step is Volmer step (1a). In alkaline media, the hydrogen evolution reaction can occur via the Heyrovsky step (1b) or the Tafel step (1c), or both, to yield H_2_. As illustrated in the following equations, there are two alternative reaction steps: Volmer step (2a) and Heyrovsky step (2b).

12 $$\begin{array}{cc}{{\text{H}}}_{2}\mathrm{O }+ e (2{\text{a}}) -& =\mathrm{ OH}- +\mathrm{ Had},\end{array}$$

13 $${{\text{H}}}_{2}\mathrm{O }+ e (2{\text{b}}) - +\mathrm{ Had }=\mathrm{ OH}- + {{\text{H}}}_{2}$$

Theoretical simulations have shown that HER activity was related to hydrogen adsorption (*H*_*ad*_). The free energy of hydrogen adsorption (Δ*GH*) is widely accepted as a descriptor for a hydrogen evolution material. Platinum appears to be the best catalyst for HER in both media which have the optimal hydrogen adsorption energy showing the highest exchange current density. It is crucial to trade-of H_ad_, hydroxy adsorption (OH_ad_), and water dissociation for HER activity in alkaline media.

In alkaline conditions, HER activity is often lower than in acidic media. This is mostly because the slow water dissociation phase slows down the reaction, resulting in a 2–3 orders of magnitude decrease in reaction rate. In industrial facilities, alkaline electrolysis is the preferred method. Electrocatalysts with high alkaline HER performance must be rationally designed with the ability to dissociate water and bind hydrogen species (Skúlason et al. 2007; Strmcnik et al. 2013).

At 700 mA/cm^2^, a 0.5-cm gap spacing, and 25% KOH, the water electrolysis efficiency is 95%. 1 kwt/h will acquire an electrolysis voltage of 1.9 V. For each mole of water, 526.316 A, or 1.9 g, of green hydrogen will be created. This can be applied at a rate of 18.65 g/h to a 752 cm^2^ Ni-Co-nanographene structure cathode by applying the Faraday’s equation. In order to produce 1 kg of green hydrogen at an electrolysis voltage of 1.9 V, 28357.55 A, or 53.88 kW/h, power is needed. Therefore, in order to bring power consumption down to the global limit of 50 kW/h, more study into improving electrode properties to serve as both cathode and anode for hydrogen generation via electrolysis is recommended. The water electrolysis efficiency is 95% at 700 mA/cm^2^, 0.5-cm gap distance, and 25% KOH. With an electrolysis voltage of 1.75 V, 1 kW/h will acquire That is, 526.316 A will be produced for every mole of water, or 1.9 g of green hydrogen, using the computation of Faraday’s equation, which yields 18.65 g/h on a 752 cm^2^ Ni-Co-nano-graphene structure cathode. At an electrolysis voltage of 1.75 V, the required power for producing one kilogram of green hydrogen is therefore 28.357 kA, or 53.88 kWh^−1^. Thus, further research into strengthening electrode characteristics to function as both cathode and anode for hydrogen generation via electrolysis is advised in order to reduce power consumption to the global limit of 50–55 kW/h (Zittel and Wurster 1996).

## Conclusion

The best conditions for the fabrication of Ni-Co-nano-graphene cathode for the synthesis of green hydrogen in a 25% KOH solution were investigated. The electrolysis efficiency was 95.6% at 700 mA/cm^2^ while electrode gap spacing was 0.5 cm and particles’ diameter in the range of 2 nm. The trace addition of nano-graphene to the electrodeposited Ni-Co thin film results in a reduction in hydrogen generation power consumption of roughly 8.1% compared to pure nickel thin film cathode. For the pure nickel thin film cathode, the power consumption was 56.212 kW/h while for the nickel cobalt thin film cathode was 54.22 kWh^−1^.

## Data Availability

All data generated or analyzed during this study are included in this published article.
